# Exposure or pandemic effect: Export boom in instant noodles from South Korea during COVID‐19

**DOI:** 10.1002/fsn3.3724

**Published:** 2023-10-08

**Authors:** Yin‐Hua Quan, Jeong‐Bin Im

**Affiliations:** ^1^ Seoul National University College of Agriculture and Life Sciences Seoul Korea; ^2^ Research Institute of Agriculture and Life Sciences Seoul National University Seoul Korea

**Keywords:** COVID‐19, exposure effect, instant noodle export, Korean wave, pandemic effect

## Abstract

Amid the pandemic, it is crucial to comprehend both people's preferred food choices and the factors influencing food consumption. This understanding not only guarantees a stable food supply but also plays a pivotal role in economic recovery during times of recession. In 2020, South Korean instant noodles experienced an unprecedented surge in exports, catapulting the nation to the forefront of the global instant noodle market. The surge in the popularity of instant noodles during the pandemic can be partly attributed to their unique characteristics. However, the South Korean film *Parasite*, which garnered global acclaim around the same time, prominently featured instant noodle products. This exposure led to an extraordinary increase in internet searches for Korean‐brand instant noodles. Therefore, this study utilized an interrupted time series model to investigate whether the surge in South Korean instant noodle exports was primarily a result of the 2019 coronavirus pandemic or the influence of the film. Our estimations indicate that the film's exposure effect predominantly explains the export boom of South Korean instant noodles.

## Graphical abstract

Even when contemplating foods well suited for pandemic consumption, the exposure effect remains a substantial factor shaping food choices.

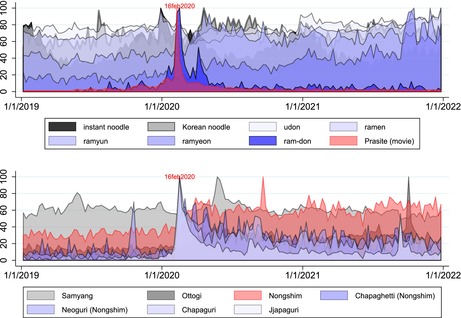

## 1 INTRODUCTION

*Parasite* made history in the realm of Asian cinema. It has been released in over 40 countries and regions since May 2019, garnering numerous prestigious film awards worldwide, including the renowned Golden Globe Awards and the Academy Awards (also known as the Oscars). In particular, the film clinched the Best Foreign Language Film award at the 77th Golden Globe Awards on January 6, 2020, and secured four major awards at the 92nd Academy Awards on February 9, 2020, notably becoming the first non‐English language film to win the Best Picture category. These accolades garnered extensive attention and received widespread coverage in the international media.

**FIGURE 1:**
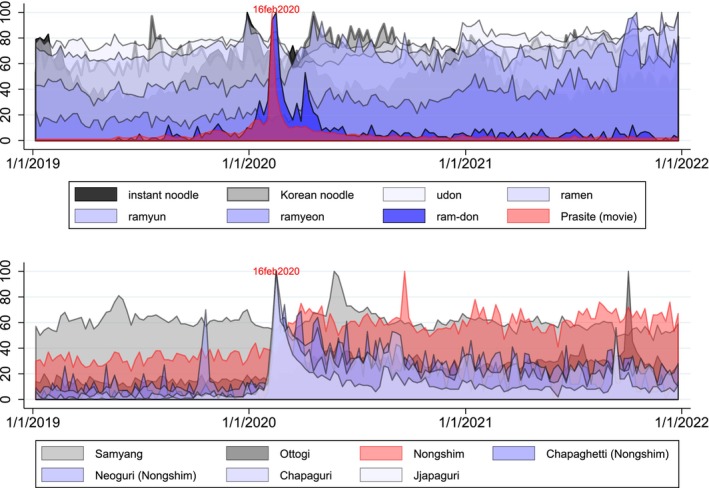
These visuals depict trends in online searches for instant noodles and related keywords worldwide, spanning from January 1, 2019, to January 1, 2022. The data was collected from Google Trends, and the *Y*‐axis is scaled from 0 to 100. A peak value of 100 signifies the highest level of popularity for the term, while 0 indicates insufficient data.

In the film, a scene impressively depicts the preparation and consumption of an instant noodle dish in South Korea. This hybrid dish is translated in the English subtitle as “ram‐don,” which is an amalgamation of “ramen” (pronounced as *ramyeon* or *ramyun*, the Korean term for instant noodles) and “udon” (borrowed from a Japanese word for a thick wheat‐flour noodle). Not only does “ram‐don” make its appearance no less than nine times within a mere 10‐min sequence, but the two brands of instant noodle products (i.e., Chapaghetti and Neoguri) are also clearly exposed to viewers. Figure 1 illustrates the internet search volume for the term “ram‐don” during mid‐2019, coinciding with the film's release. However, a significant surge in search interest began in January 2020, coinciding with the film's Golden Globes victory, and reached its zenith on February 16th of the same year, just a week after the 92nd Academy Awards.

While the exact cause of this trend remains uncertain, South Korea achieved significant success in the global instant noodle market in 2020, marked by a notable 29% increase in export value. Nongshim, the producer of the two instant noodle products featured in the film, commands a dominant domestic market share of over 55% among several companies in South Korea. Additionally, it stands as one of the largest exporters of instant noodles in South Korea. Notably, the search volume for ‘Nongshim’ has experienced a substantial surge since January 2020, surpassing that of its two primary competitors, Samyang and Ottogi. Similarly, the search volume for the names ‘Chapaguri’ and ‘Jjapaguri’ (a combination of the two products showcased in the film, namely Chapaghetti and Neoguri) has steadily increased since January 2020.

**FIGURE 2:**
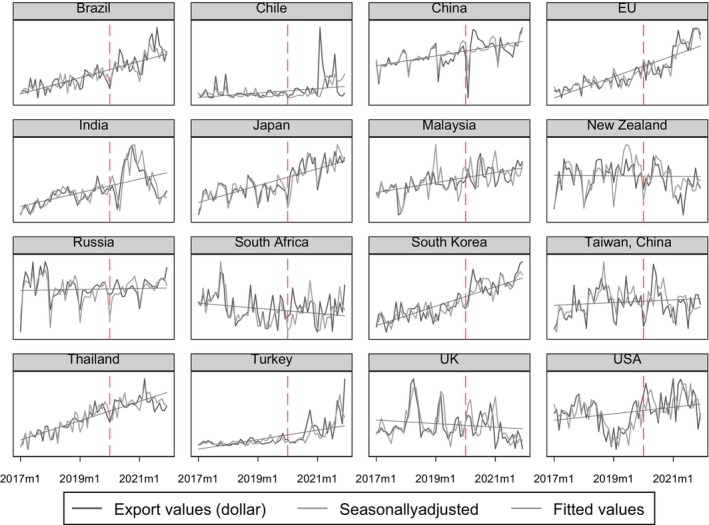
The graph illustrates the export values of instant noodles among 16 exporters from January 2017 to December 2021. The *Y*‐axis is scaled differently to accommodate the wide variation in export values among exporters. The figure highlights the trends and fluctuations in export values of instant noodles both before and after January 2020, marked by the red lines. Consequently, the *Y*‐axis has been omitted for clarity. Detailed statistics can be found in Table 1.

During a similar period, the Coronavirus disease, known as COVID‐19, emerged in late 2019, spreading rapidly across the globe and persisting to the present day. COVID‐19 prompted panic‐buying behaviors, a gradual shift toward a home‐based economy, and contributed to a global economic slowdown. The attributes of instant noodles, including their delectable taste, affordability, ease of preparation, and extended shelf life, have rendered them a suitable food choice in times of crisis. According to a study by Market Research Future (2021), COVID‐19 has presented a transformative opportunity for new brands of instant noodles within the global market. Figure 2 illustrates that the majority of exporters have shown an upward trend in the export values of instant noodles during the pandemic, with pronounced fluctuations notably occurring after 2020.

As a result, a natural question arises: Was the surge in South Korean instant noodle exports driven by the COVID‐19 pandemic or the success of the film Parasite? To address this query, the study employed an interrupted time series (ITS) analysis. A significant challenge in this study is that both interventions occurred almost simultaneously, making it challenging to pinpoint the precise factors contributing to the export boom of South Korean instant noodles. Since we lack sufficient data on instant noodle exports at the brand level, we can only make comparisons at the country level. Our hypothesis is as follows: The pandemic significantly affected the trade value of all instant noodle exporters in a similar manner, regardless of the brand, whereas the film had a positive impact on the export of South Korean instant noodle brands. This hypothesis also implies that the film's success not only boosted the exports of specific instant noodle brands but also enhanced the overall performance of the instant noodle category in the country.

Based on this fundamental assumption, the study structured the analysis as follows. Firstly, working within the confines of available data, we included only 15 additional exporters as references and applied the single‐group model of the ITS to each exporter. Our objective was to assess whether the intervention had significant effects, with a particular focus on South Korea. Additionally, we conducted a robustness test for the intervention by incorporating alternative pseudo‐start periods into the single‐group model. Secondly, we utilized the multiple‐group model of the ITS to identify comparable exporters in the context of South Korean instant noodle exports. Subsequently, we investigated whether the intervention had a more substantial impact on the export of South Korean instant noodles in comparison to comparable exporters.

## 2 APPLIED THEORY

### 2.1 Exposure and food demand

Exposure to an item causes people to develop a preference for it, and repeated exposure further increases the likelihood of such an attitude. This psychological phenomenon is called the mere‐exposure effect (Zajonc, 1968). In marketing, the mere‐exposure effect is frequently used to arouse positive feelings about a brand or a product through saturated advertising or a penetrative campaign (Baker et al., 1986). Scholars report that this effect has been particularly prevalent in the food industry (Halford et al., 2004; Harris et al., 2009; Pliner, 1982). Numerous studies have shown that exposure to televised food advertising increases consumption among children and adults (Andreyeva et al., 2011; Wong et al., 2020; Zimmerman & Shimoga, 2014).

Alternatively, modern film provides viewers with extensive sensory stimulation. Therefore, engaging with the content of the film can exert a very positive advertising effect (Riley et al., 1998). Nearly all film genres rely on food to convey important aspects about the emotions as well as the personal and cultural identities of the characters. In addition, the ethnic, religious, sexual, and philosophical aspects of narratives are also conveyed through food (Bower, 2012). Hence, it can be argued that the exposure effect facilitated by a film tends to be more encompassing. For instance, if a particular food is prominently featured in South Korean films as a carrier of the cultural symbols of South Korea, it is likely to create the impression of being closely associated with South Korea; in a narrower context, it can serve as an alternative representation of South Korean cuisine. This rationale may elucidate the stable upward trend exhibited by ‘ramyeon’ and ‘ramyun’ as alternative terms for Korean instant noodles (as depicted in Figure 1) since the onset of 2020.

### 2.2 Pandemic and food demand

Panic‐buying behavior due to the pandemic became common in many countries (Yuen et al., 2020). Panic buying typically occurs when people perceive the arrival of a disaster and stock up on daily necessities, especially food and household items (Sim et al., 2020). As demonstrated by news reports from various countries, non‐perishable foods have been particularly popular among consumers since the beginning of COVID‐19 (Kovalenko & Mazaheri, 2021).

Three years have passed since COVID‐19 broke out; during this time, the lockdowns resulted in increased working hours at home (Kramer & Kramer, 2020; Prentice et al., 2022; Rabiei et al., 2022) and led to an increase in the homebody economy (Charm et al., 2020). Both of these factors have been accompanied by an increase in home cooking (Dartnell & Kish, 2021). However, cooking requires the availability of numerous ingredients and utensils (Kovalenko & Mazaheri, 2021). Therefore, simple recipes for tasty dishes are preferred. For example, the consumption of convenience foods has significantly increased in Denmark and Germany during COVID‐19 (Janssen et al., 2021). In Italy, more frozen foods and pasta have been purchased than fresh foods during the quarantine (Leal Filho et al., 2021). The COVID‐19 outbreak has also brought the global economy to a halt (Abodunrin et al., 2020). The ongoing economic dislocation has caused income losses, which have severely impacted people belonging to low‐income brackets (Reinhart & Reinhart, 2020). Such an income shock is likely to lead to more low‐cost products being bought and consumed (Eskandari et al., 2022; Kavle et al., 2023).

According to the World Instant Noodles Association (WINA), instant noodles have become accepted as a food that meets the five core criteria, namely, delicious taste, safety, convenience, shelf life, and affordability, since its invention in 1958. In addition, nutritional value and environmental sustainability have been established as the sixth and seventh values, respectively, as principles for development in the future. These attributes lead us to conclude that instant noodles are highly suitable for consumption during a pandemic.

## 3 DATA AND METHODOLOGY

### 3.1 Data collection

The majority of the data were derived from the Korea International Trade Association (KITA), whereas the remaining data were sourced from the government websites of each exporter. These exporters are Brazil, China, India, Japan, Taiwan, Thailand, and the United States. The Harmonized Commodity Description and Coding System (HS code, which is a list of numbers used by customs to classify products) for instant noodles across these exporters varies; however, all instant noodles fall under HS190230 (Table A1). In the case of the Taiwan region, the study combined two HS codes into a single instant noodle product. The reason is that Taiwan classifies instant noodles as products, with and without meat. In addition, all exporters, except for the European Union (EU) and Japan, reported their trade values for instant noodles in U.S. dollars. Thus, data for these two exporters were converted using the average annual exchange rate from Macrotrends.net. Also, the United Kingdom (UK) left the EU on February 1, 2020, but the data were included in the EU until January of the following year, so we excluded the UK data from the EU data.

We used original and seasonally adjusted data as the outcome variables, with the rationale that seasonally adjusted data are well‐known to pose certain practical problems, whereas using unadjusted data could lead to less conclusive results (Ghysels & Perron, 1993). Seasonal adjustment was performed on the original data using the Holt–Winters multiplicative method, which is known for its robustness, ease of use, and accuracy in practice (Chatfield & Yar, 1988). Moreover, a two‐tailed variance‐comparison test and a mean‐comparison test were performed between the pre‐ and post‐intervention periods. According to the *p*‐values presented in Table 1, approximately half of the variances and means of the export values of the exporters for instant noodles are significantly different (i.e., *p* < .1) between the pre‐ and post‐intervention periods of the original and seasonally adjusted data. Lastly, the data are strongly balanced.

**TABLE 1:** Description of statistics.

Exporters	Export values (thousand dollar)—original data							Export values (thousand dollar)—seasonally adjusted						
	Obs.	Mean	SD	Min	Max	(*F*) *p*‐value^a^	(*T*) *p*‐value^b^	Obs.	Mean	SD	Min	Max	(*F*) *p*‐value^a^	(*T*) *p*‐value^b^
Brazil	60	301.88	170.30	36.62	790.40	.0010	.0000	60	292.99	144.59	75.61	627.37	.0550	.0000
Chile	60	25.03	32.57	3	199	.0000	.0039	60	19.30	17.93	2.92	71.01	.0000	.0006
China	60	24,231.34	4338.42	9520.70	33,417.32	.0096	.0000	60	24,545.05	3402.47	15,341.42	32,125.57	.8446	.0000
EU	60	27,581.72	7233.862	17,329.58	44,218.36	.0000	.0000	60	27,312.93	6793.51	18,442.03	44,486.20	.0000	.0000
India	60	1180	483.51	410	2590	.0002	.0000	60	1205.01	516.92	446.04	2611.06	.0000	.0000
Japan	60	5471.75	1443.14	2419.97	8224.61	.0675	.0000	60	5445.07	1442.85	2839.92	8833.53	.0082	.0000
Malaysia	60	6176.95	1237.27	2550	9142	.9570	.0000	60	6264.09	1417.03	2763.04	10,092.33	.2884	.0000
New Zealand	60	1636.01	523.92	232.35	2498.41	.7664	.0351	60	1739.99	553.80	491.57	2946.66	.1094	.1278
Russia	60	6387.6	1330.73	1203	9366	.0182	.8724	60	6172.43	1355.72	1740.71	8400.65	.0828	.6555
South Africa	60	1008.87	282.92	491	1739	.3472	.7027	60	963.56	289.48	519.98	1963.32	.5377	.2560
South Korea	60	42,317.63	10,911.12	22,790.77	66,654.80	.2291	.0000	60	41,667.12	9896.04	24,601.83	60,605.57	.2833	.0000
Taiwan	60	4774.82	848.65	3227	7121	.7248	.9718	60	4873.14	743.39	3082.88	7269.57	.7714	.9806
Thailand	60	26,492.98	4650.83	16,382.6	38,386.38	.0408	.0000	60	26,682.59	4932.58	17,112.3	36,202.5	.0277	.0000
Turkey	60	1083.66	1277.51	104.29	7004	.0000	.0000	60	960.94	1012.83	157.62	5367.97	.0000	.0000
UK	60	2180.8	728.77	1013	4608	.3858	.1488	60	2277.72	747.10	1138.29	4181.52	.7111	.3392
USA	60	6594.87	727.44	4882.40	8039.10	.9692	.0001	60	6537.87	667.42	5031.90	7821.15	.2340	.0000

^a^ A two‐tailed variance‐comparison test was conducted between pre‐ and post‐intervention groups; the *p*‐values are based on the *F* statistic.

^b^ A two‐tailed mean‐comparison test was conducted between pre‐ and post‐intervention groups; the *p*‐values are based on the *T* statistic.

Prior to estimation, we proposed the Phillips–Perron (PP) test and the Im–Pesaran–Shin test (IPS) to examine the presence of the unit root in the log‐transformed outcome variables. The IPS test considers not only the residual serial correlation but also the heterogeneity of error variances and dynamics across panels (Im et al., 2003). In the case where the disturbance of each panel in the IPS method is serially correlated, we used the Akaike Information Criterion to determine the optimal lags for the augmented Dickey–Fuller regression. In addition, the study accounted for time trends in both methods, and the null hypothesis is that a unit root exists.

### 3.2 Estimation method

This study applied the ITS analysis employed by Linden (2015), which is considered a desirable study design when a randomized experiment is impossible. The single‐group model (Equation 1) and multiple‐group model (Equation 2) of the ITS are as follows:

(1) $$Y_{t}=\beta_{0}+\beta_{1}T_{t}+\beta_{2}X_{t}+\beta_{3}X_{t}T_{t}+u_{t}$$

(2) $$\begin{array}{c} Y_{t}=\beta_{0}+\beta_{1}T_{t}+\beta_{2}X_{t}+\beta_{3}X_{t}T_{t}+\beta_{4}Z+\beta_{5}ZT_{t}+\beta_{6}ZX_{t}+\beta_{7}ZX_{t}T_{t}+u_{t} \end{array}$$

(3) $$\begin{array}{c} u_{t}=\rho u_{t-k}+{ɛ}_{t},t=1\cdots T\; \end{array}$$

In Equations 1 and 2, $Y_{t}$ denotes an outcome variable measured at equal time intervals. Therefore, $Y_{t}$ stands for the log‐transformed monthly export value. $T_{t}$ is the time variable, which ranges from January 2017 to December 2021 (60 months in total). $X_{t}$ represents a dummy variable that denotes the intervention (1 and 0 for post‐intervention and pre‐intervention, respectively), and January 2020 is the time of the intervention. We selected January 2020 because, according to *Timeline: WHO's COVID‐19 Response* (2022), the very first action on COVID‐19 occurred on December 31, 2019. China informed the focal point of the International Health Regulations at the WHO Western Pacific Regional Office about cases of “viral pneumonia” in Wuhan. Several health authorities from around the world contacted the WHO and requested additional information. On January 1, 2020, the WHO activated its Incident Management Support Team as part of its emergency response framework and notified partners of the Global Outbreak Alert and Response Network the following day regarding the cluster of pneumonia cases in China. In addition, January 2020 was the month when the main keyword related to the film *Parasite* began showing a strong upward trend due to the Golden Globes win.

In Equation 1, $\beta_{0}$ denotes the intercept (i.e., baseline level); $\beta_{1}$ represents the slope up to the introduction of the intervention; $\beta_{2}$ refers to changes in outcome level that occur in the period immediately after the intervention relative to the counterfactual situation; and $\beta_{3}$ pertains to differences in slopes between the pre‐ and post‐intervention periods. We ran this single‐group model for each exporter and identified significance in $\beta_{2}$ and $\beta_{3}$, but $\beta_{2}$ will be particularly noticed. This is because people may be less sensitive or fearful of COVID‐19 over time than they were at the beginning (due to the widespread availability of vaccines or the marginal effect of fear toward COVID‐19 declines over time), and also because the duration of the exposure effect of the film *Parasite* is unknown.

In Equation 2, Z is also a dummy variable, which stands for cohort membership (0 = observation group; 1 = comparison group). Accordingly, $\beta_{0}$–$\beta_{3}$ represent the observation group (e.g., South Korea), whereas the remaining coefficients represent the comparison group. $\beta_{4}$ and $\beta_{5}$ denote the differences in levels and slopes, respectively, between the observation and comparison groups; $\beta_{6}$ represents the level of differences between both groups in the period immediately after an intervention; $\beta_{7}$ indicates the slope differences between both groups in the post‐intervention period compared with the pre‐intervention period. Notably, in the multiple‐group model of the ITS, $\beta_{4}$ and $\beta_{5}$ play an essential role in determining whether both groups are equivalent in terms of the level and slope of the outcome variable at pre‐intervention. If equivalence between groups cannot be ensured, then causal inferences about the relationship between the intervention and outcomes are likely to be questionable in the multiple‐group model (Linden & Adams, 2011).

The model was estimated using ordinary least squares (OLS) regression with Newey–West standard errors in lag k as Equation 3 to account for autocorrelation, and to handle possible heteroskedasticity. In Equation 3, |ρ| < 1 and ρ is the correlation coefficient between adjacent error terms, ${ɛ}_{t}$ are independent $N\left(0,\sigma^{2}\right)$. We used OLS regression instead of the other estimation method based on an autoregressive integrated moving‐average model because OLS is more flexible and widely applicable in the context of ITS analysis (Box et al., 2015; Velicer & Harrop, 1983). Moreover, a general Cumby–Huizinga test was used to test for autocorrelation to ensure that the model was fitted with the correct autocorrelation structure (Tables A2 and A3); a maximum of approximately 10 lags was considered based on Schwert's formula (Schwert, 2002). And we used the lag with the smallest *p*‐value (if this criterion was insufficient, then we selected a larger chi‐squared value). If none of the lags reached a *p*‐value < .5, we assumed that no autocorrelation existed (Baicker & Svoronos, 2019). The final selection of lags for the models can be found in Tables A2 and A3 in the Appendix. Finally, the statistical analysis tool used for the estimation is STATA/MP 17.0.

## 4 RESULTS

As shown in Table 2, with the exception of the original data from India, the rest of the panel rejects the null hypothesis of the existence of a unit root at the 0.1 significance level for the PP and IPS tests. In addition, the majority of the panel rejects the null hypothesis at the 0.01 significance level. After first‐order differencing, the original data from India also reject the null hypothesis. This finding suggests that it is difficult to say that the data are not stationary.

**TABLE 2:** Result of Phillips–Perron test and Im–Pesaran–Shin test for the presence of unit root.

Method	Exporters	Log‐transformed export values of instant noodles	
		Original data	Seasonally adjusted data
PP	Brazil	0.0000 (−8.288)***	0.0000 (−8.939)***
	Chile	0.0000 (−5.938)***	0.0197 (−3.743)**
	China	0.0000 (−6.973)***	0.0000 (−7.698)***
	EU	0.0001 (−5.208)***	0.0000 (−5.335)***
	India	0.0000 (−7.974)***	0.0814 (−3.215)*
	Japan	0.0000 (−5.736)***	0.0004 (−4.868)***
	Malaysia	0.0006 (−4.733)***	0.0001 (−5.149)***
	New Zealand	0.0000 (−5.743)***	0.0000 (−5.695)***
	Russia	0.0000 (−9.320)***	0.0000 (−6.641)***
	South Africa	0.0000 (−6.304)***	0.0018 (−4.448)***
	South Korea	0.0000 (−5.794)***	0.0000 (−5.739)***
	Taiwan	0.0000 (−5.695)***	0.0023 (−4.381)***
	Thailand	0.0000 (−5.880)***	0.0000 (−7.183)***
	Turkey	0.0009 (−4.645)***	0.0058 (−4.127)***
	UK	0.0089 (−3.996)***	0.0241 (−3.675)**
	USA	0.0019 (−4.441)***	0.0160 (−3.812)**
IPS	All panels	0.0000 (−13.8953)***	0.0000 (−9.8092)***

*Note*: The original data from India were the first‐order differenced series. The Phillips–Perron tau test statistic in the parentheses for the PP test, and a standardized t‐bar statistic is enclosed in parentheses for the IPS test.

*p < .1; **p < .05; ***p < .01.

**FIGURE 3:**
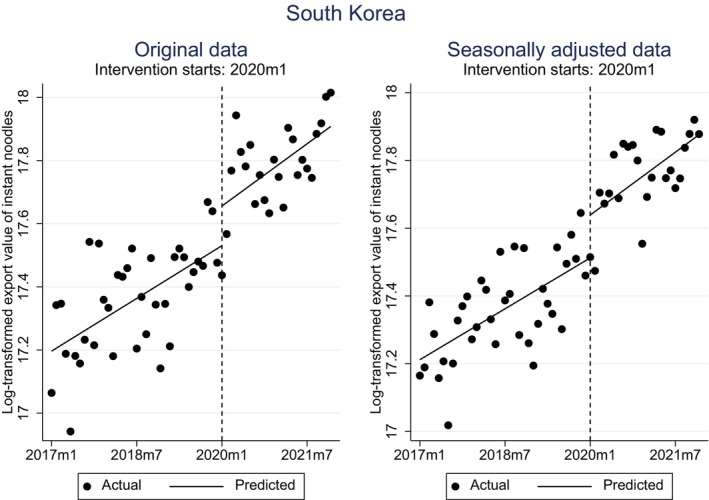
Outline of the single‐group model of the ITS for South Korea, that is, original and seasonally adjusted data.

**FIGURE 4:**
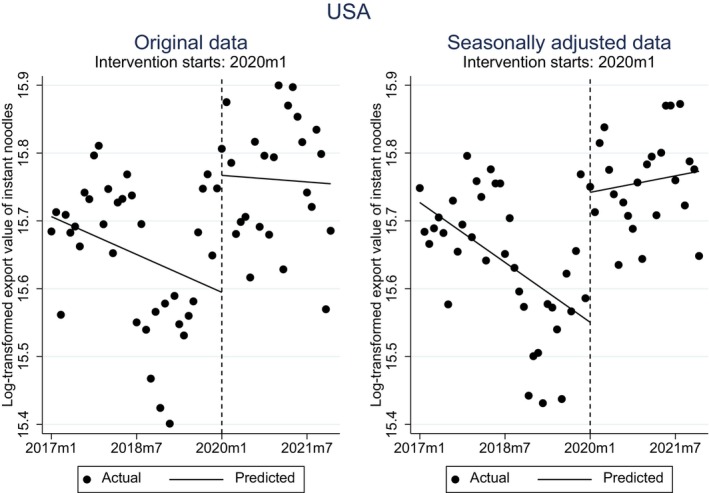
Outline of the single‐group model of the ITS for the United States, that is, original and seasonally adjusted data.

Tables 3 and 4 present the results of the single‐group model of the ITS for the original and seasonally adjusted data, respectively. At the time of the intervention, South Korea exhibited a significant level change in both tables (*p* < .1). Figure 3 clearly depicts such a change. Interestingly, we found that the United States of America (USA) also had a significant level change in the first month of the intervention in both tables (also see Figure 4). In contrast, the remaining 14 exporters displayed no change or a negative change in level at the time of intervention. This outcome is both surprising and invigorating, considering Nongshin's established factories in the United States and its ongoing expansion initiatives. We will delve deeper into the specifics in the upcoming section.

**TABLE 3:** Result of the single‐group model of the ITS for original data.

Exporters	Log‐transformed export value of instant noodles (intervention point: January 2020)			
	Level change ($\beta_{2}$)	Pre‐intervention slop ($\beta_{1}$)	Post‐intervention slop ($\beta_{1}+\beta_{3}$)	Difference in slope ($\beta_{3}$)
Brazil	−0.07 (0.14)	0.03 (0.00)***	0.03 (0.01)***	0.00 (0.01)
Chile	0.15 (0.48)	0.01 (0.01)	0.04 (0.04)	0.04 (0.04)
China	−0.03 (0.16)	0.01 (0.00)**	0.01 (0.01)	0.00 (0.01)
EU	−0.03 (0.00)	0.01 (0.00)***	0.02 (0.00)***	0.02 (0.00)***
India	0.05 (0.13)	−0.00 (0.00)	−0.00 (0.01)	−0.00 (0.10)
Japan	0.16 (0.10)	0.01 (0.00)*	0.01 (0.01)**	0.01 (0.01)
Malaysia	0.03 (0.07)	0.01 (0.00)**	0.01 (0.00)	−0.00 (0.01)
New Zealand	−0.22 (0.17)	0.01 (0.01)*	−0.02 (0.01)	−0.03 (0.02)*
Russia	−0.12 (0.11)	0.00 (0.01)	0.01 (0.00)***	0.01 (0.01)
South Africa	0.21 (0.14)	−0.01 (0.00)***	0.00 (0.01)	0.01 (0.01)
South Korea	0.13 (0.06)**	0.01 (0.00)***	0.01 (0.00)***	0.00 (0.00)
Taiwan	−0.13 (0.12)	0.01 (0.00)***	−0.00 (0.01)	−0.01 (0.01)
Thailand	−0.01 (0.04)	0.01 (0.00)***	0.00 (0.00)	−0.01 (0.00)***
Turkey	−0.06 (0.30)	0.03 (0.10)**	0.08 (0.01)***	0.06 (0.02)***
UK	0.20 (0.20)	0.00 (0.00)	−0.03 (0.01)***	−0.03 (0.01)***
USA	0.17 (0.07)**	−0.00 (0.00)	−0.00 (0.00)	0.00 (0.00)

*Note*: Newey–West standard errors are enclosed in parentheses.

*p < .1; **p < .05; ***p < .01.

**TABLE 4:** Result of the single‐group model of the ITS for seasonally adjusted data.

Exporters	Log‐transformed export value of instant noodles (intervention point: January 2020)			
	Level change ($\beta_{2}$)	Pre‐intervention slop ($\beta_{1}$)	Post‐intervention slop ($\beta_{1}+\beta_{3}$)	Difference in slope ($\beta_{3}$)
Brazil	−0.10 (0.10)	0.03 (0.00)***	0.03 (0.01)***	−0.00 (0.01)
Chile	−0.55 (0.23)**	−0.02 (0.01)*	0.12 (0.01)***	0.14 (0.01)***
China	−0.08 (0.03)**	0.01 (0.00)***	0.01 (0.00)***	0.00 (0 .00)
EU	0.01 (0.06)	0.01 (0.00)***	0.02 (0.00)***	0.01 (0.00)***
India	0.19 (0.23)	0.02 (0.00)***	−0.01(0.01)	−0.03 (0 .01)**
Japan	−0.04 (0.11)	0.01 (0.00)**	0.02 (0.01)***	0.01 (0.01)*
Malaysia	0.11 (0.11)	0.01 (0.00)**	−0.00 (0.00)	−0.01 (0.01)
New Zealand	−0.30 (0.22)	0.02 (0.01)**	−0.01 (0.01)	−0.03 (0.01)*
Russia	−0.10 (0.14)	−0.00 (0 .00)	0.02 (0.01)**	0 .02 (0.01)**
South Africa	0.02 (0.22)	−0.01 (0.01)**	0.01 (0.01)	0.03 (0.01)***
South Korea	0.13 (0.07)*	0.01 (0.00)***	0.01 (0.00)***	0.00 (0.00)
Taiwan	−0.11 (0.09)	0.01 (0.00)***	−0.00 (0.00)	−0.01 (0.01)**
Thailand	−0.04 (0.05)	0.01 (0.00)***	0.01 (0.00)*	−0.01 (0.00)**
Turkey	−0.40 (0.22)*	0.03 (0.01)***	0.09 (0.01)***	0.07 (0.01)***
UK	0.23 (0.16)	0.00 (0.01)	−0.03 (0.01)***	−0.03 (0.01)***
USA	0.19 (0.05)***	−0.00 (0.00)**	0.00 (0.00)	0.01 (0.00)**

*Note*: Newey–West standard errors are enclosed in parentheses.

*p < .1; **p < .05; ***p < .01.

Tables 5 and 6 show the results of the multiple‐group model of the ITS for the original and seasonally adjusted data, respectively. It is important to note that the baseline level before the intervention is significantly lower for all exporters than for South Korea, in both the original and seasonally adjusted data. In essence, this implies that the export performance of the 15 other exporters was incomparable to that of South Korea in the realm of instant noodle exports prior to the intervention. Likewise, we identified a single exporter, Russia, which could be compared to the United States due to similar baseline and slope values for instant noodle exports in both the original and seasonally adjusted data prior to the intervention (*p* > .10). In Russia, there are manufacturing facilities associated with another Korean instant noodle brand, which serves as the primary competitor to Nongshim. These competitors have demonstrated impressive sales performance in the European market. Once again, we will reserve further discussion of this topic for the upcoming section.

**TABLE 5:** Result of the ITS multiple‐group model for original data.

Comparison groups	South Korea as the observation group		USA as the observation group	
	Difference in level before intervention ($\beta_{4}$)	Difference in slope before intervention ($\beta_{5}$)	Difference in level before intervention ($\beta_{4}$)	Difference in slope before intervention ($\beta_{5}$)
Brazil	5.63 (0.08)***	−0.02 (0.00)***	4.13 (0.09)***	−0.03 (0.00)***
Chile	7.90 (0.19)***	0.00 (0.01)	6.41 (0.13)***	−0.01 (0.01)
China	0.37 (0.07)***	0.00 (0.00)	−1.10 (0.04)***	−0.01 (0.00)***
EU	0.39 (0.04)***	0.00 (0.00)	−1.10 (0.04)***	−0.01 (0.00)***
India	17.12 (0.08)***	0.01 (0.00)***	15.63 (0.05)***	−0.00 (0.00)
Japan	1.99 (0.08)***	0.00 (0.00)	0.50 (0.08)***	−0.01 (0.00)**
Malaysia	1.82 (0.10)***	0.00 (0.00)	0.33 (0.09)***	−0.01 (0.00)***
New Zealand	3.09 (0.17)***	−0.00 (0.01)	1.60 (0.17)***	−0.02 (0.01)**
Russia	1.62 (0.15)***	0.01 (0.01)	0.13 (0.15)	−0.01 (0.01)
South Africa	3.19 (0.09)***	0.02 (0.00)***	1.70 (0.10)***	0.01 (0.00)*
South Korea	–	–	−1.49 (0.05)***	−0.01 (0.00)***
Taiwan	1.96 (0.07)***	0.00 (0.00)	0.47 (0.06)***	−0.01 (0.00)***
Thailand	0.46 (0.05)***	−0.00 (0.00)*	−1.03 (0.04)***	−0.02 (0.00)***
Turkey	4.82 (0.25)***	−0.02 (0.10)	3.32 (0.28)***	−0.03 (0.01)***
UK	2.60 (0.10)***	0.01 (0.00)*	1.11 (0.09)***	−0.00 (0.00)
USA	1.49 (0.05)***	0.01 (0.00)***	–	–

*Note*: Newey–West standard errors are enclosed in parentheses.

*p < .1; **p < .05; ***p < .01.

**TABLE 6:** Result of the multiple‐group model of the ITS for seasonally adjusted data.

Comparison groups	South Korea as the observation group		USA as the observation group	
	Difference in level before intervention ($\beta_{4}$)	Difference in slope before intervention ($\beta_{5}$)	Difference in level before intervention ($\beta_{4}$)	Difference in slope before intervention ($\beta_{5}$)
Brazil	5.57 (0.06)***	−0.02 (0.00)***	4.09 (0.06)***	−0.03 (0.00)***
Chile	7.61 (0.16)***	0.03 (0.01)***	6.12 (0.16)***	0.01 (0.10)
China	0.40 (0.04)***	0.00 (0.00)	−1.09 (0.04)***	−0.01 (0.02)***
EU	0.39 (0.05)***	0.00 (0.00)	−1.20 (0.04)***	−0.01 (0.00)***
India	3.86 (0.09)***	−0.01 (0.00)***	2.38 (0.09)***	−0.03 (0.00)***
Japan	2.02 (0.06)***	0.00 (0.00)	0.53 (0.66)***	−0.01 (0.00)***
Malaysia	1.83 (0.08)***	0.00 (0.00)	0.34 (0.79) ***	−0.01 (0.00)***
New Zealand	3.16 (0.18)***	−0.01 (0.01)	1.67 (0.18)***	−0.02 (0.01)***
Russia	1.60 (0.09)***	0.10 (0.00)**	0.11 (0.09)	−0.00 (0.00)
South Africa	3.20 (0.11)***	0.02 (0.01)***	1.72 (0.11)***	0.01 (0.01)
South Korea	–	–	−1.48 (0.04)***	−0.01 (0.00)***
Taiwan	1.98 (0.06)***	−0.00 (0.00)	0.49 (0.06)***	−0.01 (0.00)***
Thailand	0.48 (0.04)***	−0.01 (0.00)***	−1.01 (0.04)***	−0.02 (0.00)***
Turkey	4.69 (0.16)***	−0.02 (0.01)**	3.21 (0.16)***	−0.03 (0.01)***
UK	2.61 (0.11)***	0.01 (0.01)	1.13 (0.11)***	−0.01 (0.10)
USA	1.48 (0.04)***	0.01 (0.00)***	–	–

*Note*: Newey–West standard errors are enclosed in parentheses.

*p < .1; **p < .05; ***p < .01.

Since there is no exporter of instant noodles comparable to South Korea, we do not show the result of the multiple‐group model in detail. However, we further conducted a robustness test for the intervention period in the single‐group model for South Korea and the United States (Tables A4 and A5). We chose the median timing of the pre‐intervention period (Imbens & Lemieux, 2008; Linden, 2015), i.e., June 2018 and July 2018 as pseudo‐start period. At the pseudo‐star periods, both exporters show a significant negative‐level change and no slope change.

Table 7 displays all coefficient results of the United States' multiple‐group model of the ITS by limiting the choice of comparison group to Russia, which is the only exporter comparable to the United States for exports of instant noodles. Specifically, in this table, the study intends to identify the difference in the level immediately after intervention between the United States and Russia (i.e., $\beta_{6}$). The United States increased exports of instant noodles by about 30% during the first month of the intervention compared to Russia (*p* < 0.05).

**TABLE 7:** Results of the ITS multiple‐group model between the USA (observation group) and Russia (comparison group).

Coefficients	Interpretation	Original data	Seasonally adjusted data
*β*_0_	Intercept	15.58 (0.15)***	15.61 (0.09)***
*β*_1_	Pre‐intervention slop of Russia	0.00 (0.01)	−0.00 (0.00)
*β*_2_	Level change in Russia	−0.12 (0.09)	−0.10 (0.14)
*β*_3_	Difference in slope in Russia	0.01 (0.01)	0.02 (0.01)***
*β*_4_	Difference in level before intervention between the USA and Russia	0.13 (0.15)	0.11 (0.09)
*β*_5_	Difference in slope before intervention between the USA and Russia	−0.01 (0.01)	−0.00 (0.00)
*β*_6_	Difference in level immediately after intervention between the USA and Russia	0.30 (0.11)***	0.29 (0.15)**
*β*_7_	Difference in slope after intervention compared with pre‐intervention between the USA and Russia	−0.01 (0.01)	−0.01 (0.01)*
*β*_1_ + *β*_3_ + *β*_5_ + *β*_7_	Post‐intervention trends in the USA	−0.00 (0.00)	0.00 (0.00)
*β*_1_ + *β*_3_	Post‐intervention trends in Russia	0.01 (0.00)***	0.02 (0.01)***
*β*_5_ + *β*_7_	Differences in post‐intervention trends between the USA and Russia	−0.01 (0.00)**	−0.01 (0.01)**

*Note*: Newey–West standard errors are enclosed in parentheses.

*p < .1; **p < .05; ***p < .01.

## 5 DISCUSSION

Based on the estimation results, only South Korea and the United States demonstrated a positive impact at the time of intervention, while other exporters showed non‐significant or negative effects. In essence, the immense success of the movie *Parasite* exerted a positive exposure effect on the export of instant noodles, whereas COVID‐19 may have initially had a negative impact during the intervention or even in the long term to meet increased domestic demand.

Nevertheless, why was the export of instant noodles from the USA also believed to be influenced by the film? According to information confirmed by Nongshim, it established its first instant noodle factory in the United States in 1994, which also produces the two noodle products featured in the film. Nongshim's factory in the United States mainly serves the North American market, while at the same time playing a role in accelerating its entry into South America. Because both award ceremonies were held in the United States, North America may be more affected than other regions. This conjecture could be supported by the global search data in Google Trends, which was categorized by region. Furthermore, corroborative evidence in Table A6 reveals that the majority of U.S. instant noodles are exported to countries in the Americas. Canada, for instance, constitutes nearly 90% of total U.S. instant noodle exports. From 2017 to 2019, Canada's share experienced a decline, reaching its lowest point in recent years at 84.4%, followed by a rebound from 2020 to 2022. Notably, Mexico emerged as the second‐largest importer of U.S. instant noodles in 2022.

According to Nongshim's Annual Report for 2020, the company witnessed remarkable sales growth, achieving a 33% increase compared to the previous year at its U.S. factory. Notably, 13.1% of these sales were exported to Canada. Furthermore, Nongshim's overseas manufacturing facilities are currently only located in the United States and China, with the U.S. branch undergoing rapid expansion. In April 2022, a second factory in the United States commenced operations and is poised to become the nation's largest instant noodle manufacturer. Insights from Nongshim Company indicate that Nongshim holds a prominent position among instant noodle brands in the U.S., boasting a market share of approximately 25% and representing 80% of South Korean instant noodle brands in the U.S. market.

Interestingly, as the only comparable exporter of instant noodles to the United States, Russia also has factories for a South Korean instant noodle brand called Paldo. Since entering the Russian market in 1991, Paldo Lunchbox has established itself as the “national instant noodle” in the region. As Paldo ranks first in the region in rating the quality of instant noodles, its popularity is increasing along with the credibility of Russian consumers. And along with the effect of the film *Parasite*, the exports of instant noodles from the United States were significantly higher than Russia's immediately after the intervention. However, it is worth noting that exposure effects may not have a lasting impact, as the difference in slope after the intervention, compared to the pre‐intervention period, between the USA and Russia is significantly reduced by one percentage point in the seasonally adjusted data.

Why, then, did the Nongshim factory in China not experience significant growth in Chinese instant noodle exports following the intervention? This study posits several factors. First, the COVID‐19 pandemic led to a substantial increase in domestic demand for instant noodles in China, as it was among the first countries to implement lockdown policies. According to estimated data from WINA (2021), China consumed 46,350 million servings of instant noodles in 2020, marking an 11.82% increase. However, this was only a modest 2.48% average increase from 2017 to 2019, explaining the significant decline in China's export of instant noodles in the seasonally adjusted data at the beginning of the intervention. Second, logistical constraints resulting from the pandemic may have contributed to China's decline in instant noodle exports. In contrast, the USA did not enforce lockdown policies at the time, and instant noodles were not as popular among American consumers as in China. Consequently, instant noodles produced in the United States faced less pressure to meet domestic demand, making them more readily available for export to North American markets compared to China, even during the pandemic. Third, unlike the USA, South Korea and China are geographically proximate. South Korea's Nongshim factories cater to global export supply, while China's primarily focus on the Chinese domestic market. Additionally, South Korea boasts one of the world's highest per capita consumption rates of instant noodles, and COVID‐19 was well controlled during the early stages of the global outbreak. This suggests that the pandemic had a relatively minor impact on domestic instant noodle demand in South Korea. In essence, the negative influence of COVID‐19 on Korean instant noodle exports was sufficiently offset by the export demand generated by the film's exposure. Fourth, China hosts numerous instant noodle brands with high production volumes. Consequently, even with the influence of film exposure, Nongshim's production in China was insufficient to significantly impact the total export volume of Chinese instant noodles.

Lastly, for a more robust discussion, it would be desirable if the data allowed us to use brand‐level data or add more exporters as references. And for future analyzes of the impact of a pandemic on food exports, we suggest that canned or jarred foods and frozen packaged foods are better subjects for study. This is because they are consumed on a larger scale and have a longer market history.

### 5.1 Implications

The exposure effect of the film *Parasite* implies that the development of entertainment industries with rich cultural content could be helpful for food sales. And it is relatively easy to imagine that instant noodles, as a representative food product of South Korea, are also affected by other exposure and ripple effects because the output of the Korean wave has been extremely strong worldwide in recent years. A look at the top ten importing countries for instant noodles in South Korea and the United States (Tables A6 and A7) shows that the importing countries from South Korea are quite geographically diversified, while the United States is concentrated in one particular country. Instant noodles as one of the main representatives of Korean cultural content, the ranking of importers or the value of imports may reflect the degree of influence of the Korean wave on the local environment.

In short, the increase in the consumption of cultural content is accompanied by an increase in the consumption of byproducts. Hollywood films seem to have demonstrated this tendency impressively. For example, since the film *E.T*. brought Reese's Pieces (a type of peanut butter candy) a huge sales success, manufacturers have been spending thousands of dollars to place their products in the films (Snyder, 1992). Indeed, many factors influence food consumption, that is, biologically by hunger or taste, economically by price or availability, physically by education or cooking skills and time, psychologically by mood, socially by culture or family, and attitude by religion, among others (The European Food Information Council, 2006). Although these influencing factors vary individually, instant noodles do not seem to conflict with them excessively. In other words, instant noodles are one of those items that are very suitable to place in films to increase their consumption worldwide.

An award‐winning movie is not controllable by either government or industry, but evidence has shown that even low‐cost social media can enhance business performance (Zhang et al., 2021). With the advent of short videos nowadays, an increasing number of food manufacturers are incorporating products into video clips or using food influencers to increase awareness of their products. One of the reasons is that short videos demonstrate products in a very specific and direct manner. Compared to this marketing mode, a delay may be noted in the exposure effect brought by film. This delay may be caused by the different release times in different regions or the theatrical boom triggered by film awards. But either way, the point is that the film needs to be successful enough, either at the box office or through word of mouth, to have a satisfactory exposure effect. As mentioned in Section 2.1, food is used in films as cultural symbols that not only influence a particular exposed commodity but also drive the development and prosperity of related industries in the film represented by a country. Therefore, the exposure effect of a product through a successful film could be more lasting and comprehensive than video clips.

On the other hand, we must realize that the great success of the film *Parasite* is not accidental. We can say that it is the result of the successful reform policy of the Korean film industry. Especially for an export‐oriented country like South Korea, close cooperation with the cultural industry helps strengthen the national economy. Therefore, this is inseparable from the long‐term efforts of South Korea government and people from all walks of life. Lee (2021) pointed out that the success of film *Parasite* not only brought great commercial success but also provided South Korea a strategic nation branding opportunity to strengthen soft power and cultural diplomacy.

Last but not least, the overall outlook for food security and nutrition is expected to continue to deteriorate due to COVID‐19. Food insecurity may also occur in countries and populations that were not previously affected (Chichaibelu et al., 2021). In fact, WINA members have been very active for years in providing instant noodles as food aid to disaster‐affected areas. After the outbreak of COVID‐19, thousands of instant noodles were donated to various areas to ensure an effective and sustainable food supply. However, these areas are seemingly limited to places where instant noodles have a high acceptance rate. If more people in different regions accept instant noodles as a common food through certain channels (e.g., media exposure), then instant noodles could become an ideal food aid for emergency relief to combat hunger on a larger scale.

## 6 CONCLUSIONS

This study employed the ITS model to examine the factors behind the surge in instant noodle exports from South Korea during the COVID‐19 pandemic. The findings suggest that the export upswing was primarily driven by the film ‘Parasite's’ exposure effect. In essence, the film's influence overshadowed the pandemic's impact on instant noodle exports. This pattern not only shed light on diverse food preferences across markets but also underscored that the COVID‐19 pandemic did not necessarily yield a positive effect on instant noodle exports. In fact, the opposite may hold true.

## AUTHOR CONTRIBUTIONS

**Yin‐Hua Quan:** Conceptualization (lead); data curation (lead); formal analysis (lead); investigation (equal); software (lead); visualization (lead); writing – original draft (lead). **Jeong‐Bin Im:** Investigation (equal); project administration (lead); resources (lead); supervision (lead); validation (lead); writing – review and editing (lead).

## FUNDING INFORMATION

This research received no specific grant from any funding agency in the public, commercial, or not‐for‐profit sectors.

## CONFLICT OF INTEREST STATEMENT

The authors have declared no conflict of interest.

## Data Availability

The data that support the findings of this study are available from the corresponding author upon reasonable request.

## Appendix

**TABLE A1:** Data source.

Exporters	Name of the HS code	HS code	Data source	Exchange rate conversion^a^
Brazil	Pasta nes	HS 190230	Comex Stat	–
Chile	Pasta, cooked or otherwise prepared (excl. stuffed)	HS 190230	KITA	–
China	Instant noodle	HS 19023030	Customs Statistics	–
EU	Dried	HS 19023010	KITA	Euro to U.S. dollar
India	Dried other pasta	HS 19023010	Department of Commerce, Ministry of Commerce and Industry	–
Japan	Instant ramen and other instant noodles	HS 190230100	Trade Statistics of Japan, Ministry of Finance	Yen to U.S. dollar
Malaysia	Pasta nes	HS 190230	KITA	–
New Zealand	Other pasta	HS 190230	KITA	–
Russia	Pasta nes	HS 190230	KITA	–
South Africa	Other pasta	HS 190230	KITA	–
South Korea	Ramen	HSK 1902301010	KITA	–
Taiwan	Instant noodles, containing meat; Instant noodles, not containing meat	HS 19023010107; HS 19023010205	CPT Single Window	–
Thailand	Pasta nes	HS 190230	Trade Thailand	–
Turkey	Dried, prepared pasta (excl. stuffed)	HS 19023010	KITA	–
UK	Dried	HS 19023010	KITA	–
USA	Pasta, nesoi	HS 1902300060	US Census	–

^a^ Annual average exchange rates used data from Macrotrends.net.

**TABLE A2:** Result of the Cumby–Huizinga test for autocorrelation in the ITS single‐group model.

Exporters	Log‐transformed export values of instant noodles—original data		Log‐transformed export values of instant noodles—seasonally adjusted data	
	Lags	*p*‐Value (Chi^2^)	Lags	*p*‐Value (Chi^2^)
Brazil	6	.0318 (4.607)**	6	.0004 (12.656)***
Chile	3	.0300 (4.710)**	6	.0031 (8.733)***
China	0	–	9	.0302 (4.697)**
EU	7	.0146 (5.959)**	10	.0035 (8.509)***
India	2	.0212 (5.310)**	1	.0000 (20.555)***
Japan	0	–	8	.0026 (9.094)***
Malaysia	1	.0018 (9.794)***	1	.0180 (5.592)**
New Zealand	6	.0075 (7.154)***	6	.0019 (9.610)***
Russia	0	–	0	–
South Africa	0	–	6	.0000 (18.112)***
South Korea	10	.0421 (4.133)**	4	.0174 (5.651)**
Taiwan	1	.0331 (4.539)**	1	.0012 (10.511)***
Thailand	0	–	5	.0201 (5.401)**
Turkey	9	.0125 (6.243)**	1	.0179 (5.606)**
UK	1	.0012 (10.529)***	1	.0000 (18.974)***
USA	2	.0011 (10.700)***	8	.0002 (13.893)***

***p* < .05; ****p* < .01.

**TABLE A3:** Result of the Cumby–Huizinga test for autocorrelation in the ITS multiple‐group model.

Data	Comparison groups	South Korea as the observation group		USA as the observation group	
		Lags	*p*‐Value (Chi^2^)	Lags	*p*‐Value (Chi^2^)
Original	Brazil	6	.0082 (6.991)***	6	.0027 (8.985)***
	Chile	3	.0023 (9.278)***	7	.0058 (7.600)***
	China	0	–	0	–
	EU	10	.0332 (4.534)**	10	.0005 (12.186)***
	India	2	.0073 (7.186)***	8	.0264 (4.928)**
	Japan	10	.0229 (5.174)**	10	.0107 (6.522)**
	Malaysia	1	.0003 (12.949)***	1	.0000 (18.346)***
	New Zealand	6	.0007 (11.405)***	6	.0002 (13.774)***
	Russia	10	.0108 (6.505)**	10	.0072 (7.228)***
	South Africa	0	–	6	.0205 (5.366)**
	South Korea	–	–	10	.0005 (12.097)***
	Taiwan	1	.0087 (6.890)***	1	.0014 (10.248)***
	Thailand	4	.0217 (5.273)**	8	.0010 (10.774)***
	Turkey	1	.0006 (11.847)***	9	.0004 (12.547)***
	UK	1	.0000 (16.998)***	1	.0000 (20.253)***
	USA	10	.0005 (12.097)***	–	–
Seasonally adjusted	Brazil	6	.0000 (19.654)***	6	.0000 (22.231)***
	Chile	6	.0001 (16.374)***	6	.0000 (16.925)***
	China	4	.0224 (5.213)**	9	.0005 (12.134)***
	EU	10	.0327 (4.563)**	10	.0000 (20.640)***
	India	1	.0000 (35.088)***	1	.0000 (39.212)***
	Japan	8	.0002 (13.733)***	8	.0000 (19.613)***
	Malaysia	1	.0025 (9.169)***	1	.0006 (11.897)***
	New Zealand	6	.0000 (16.740)***	6	.0000 (17.948)***
	Russia	10	.0209 (5.337)**	10	.0074 (7.165)***
	South Africa	6	.0000 (27.711)***	6	.0000 (31.478)***
	South Korea	–	–	8	.0012 (10.532)***
	Taiwan	1	.0003 (13.058)***	1	.0000 (19.947)***
	Thailand	4	.0049 (7.931)***	8	.0006 (11.828)***
	Turkey	1	.0011 (10.650)***	1	.0007 (11.387)***
	UK	1	.0000 (31.950)***	1	.0000 (36.148)***
	USA	8	.0012 (10.532)***	–	–

Note: ***p* < 0.05; ****p* < 0.01.

**TABLE A4:** Robustness test for intervention points in a single‐group model of South Korea.

Intervention point	Original data		Seasonally adjusted data	
	Level change ($\beta_{2}$)	Difference in slope ($\beta_{3}$)	Level change ($\beta_{2}$)	Difference in slope ($\beta_{3}$)
2018 m6	−0.12 (0.05)***	−0.00 (0.00)	−0.05 (0.07)	0.00 (0.00)
2018 m7	−0.19 (0.05)***	−0.00 (0.00)	−0.10 (0.05)**	0.00 (0.00)

***p* < .05; ****p* < .01.

**TABLE A5:** Robustness test for intervention point in a single‐group model of the United States.

Intervention point	Original data		Seasonally adjusted data	
	Level change ($\beta_{2}$)	Difference in slope ($\beta_{3}$)	Level change ($\beta_{2}$)	Difference in slope ($\beta_{3}$)
2018 m6	−0.20 (0.05)***	0.00 (0.00)	−0.18 (0.05)***	0.00 (0.00)
2018 m7	−0.21 (0.04)***	0.00 (0.00)	−0.20 (0.05)***	0.00 (0.00)

***p* < .05; ****p* < .01.

**TABLE A6:** Top 10 importers of instant noodles from the United States from 2017 to 2022.

Exporters	USA									
2017	Canada	Norway	Brazil	Bahamas	Mexico	China	United Arab Emirates	Philippines	Dominican Republic	South Korea
% of instant noodle exports	91.4	3.2	1.8	0.7	0.5	0.3	0.2	0.2	0.2	0.1
2018	Canada	Norway	Brazil	Dominican Republic	Mexico	Bahamas	Trinidad and Tobago	United Arab Emirates	Venezuela	Japan
% of instant noodle exports	86.9	3.7	2.6	1.8	0.9	0.7	0.6	0.3	0.2	0.2
2019	Canada	Norway	Mexico	Jamaica	Dominican Republic	Trinidad and Tobago	Bahamas	Brazil	United Arab Emirates	Guyana
% of instant noodle exports	84.4	3.1	2.9	2.6	1.6	1.3	1.1	0.3	0.3	0.2
2020	Canada	Jamaica	Bahamas	Mexico	Dominican Republic	Trinidad and Tobago	Venezuela	Brazil	Panama	Japan
% of instant noodle exports	89.2	2.4	1.6	1.3	0.9	0.6	0.5	0.3	0.2	0.2
2021	Canada	Jamaica	Dominican Republic	Mexico	Bahamas	Trinidad and Tobago	Venezuela	Panama	Guyana	South Korea
% of instant noodle exports	89.1	2.7	1.5	1.4	1.4	1.0	0.4	0.2	0.2	0.1
2022	Canada	Mexico	Bahamas	Jamaica	Trinidad and Tobago	Argentina	Venezuela	Dominican Republic	UK	British Virgin Islands
% of instant noodle exports	90.9	2.4	2.1	0.7	0.4	0.3	0.3	0.2	0.2	0.2

*Data source*: KITA.

**TABLE A7:** Top 10 importers of instant noodles from South Korea from 2017 to 2022.

Exporters	South Korea									
2017	China	USA	Japan	Taiwan	Thailand	Malaysia	Australia	Indonesia	Philippines	Vietnam
% of instant noodle exports	27.1	10.8	6.7	5.5	5.0	4.4	4.3	3.8	3.6	3.6
2018	China	USA	Japan	Taiwan	Malaysia	Thailand	Australia	Indonesia	Vietnam	Hong Kong
% of instant noodle exports	22.6	12.2	7.7	4.9	4.8	4.7	4.6	4.4	3.6	3.4
2019	China	USA	Japan	Indonesia	Taiwan	Australia	Vietnam	Thailand	Malaysia	Philippines
% of instant noodle exports	26.6	11.5	7.2	4.7	4.3	4.1	4.1	4.1	3.5	3.1
2020	China	USA	Japan	Thailand	Philippines	Taiwan	Australia	Malaysia	UK	Hong Kong
% of instant noodle exports	24.7	13.6	9.0	4.4	3.9	3.9	3.5	3.2	2.9	2.9
2021	China	USA	Japan	Taiwan	Thailand	Philippines	Malaysia	Australia	Netherlands	UK
% of instant noodle exports	22.2	12.0	9.7	4.7	4.4	4.2	4.1	3.1	2.9	2.8
2022	China	USA	Japan	Philippines	Thailand	Taiwan	Netherlands	Malaysia	Australia	UK
% of instant noodle exports	24.7	10.0	7.9	4.1	4.0	4.0	3.9	3.9	3.5	3.2

*Data source*: KITA.
